# A geocoded dataset of primary health care clinics in Brazil

**DOI:** 10.1016/j.dib.2025.112085

**Published:** 2025-09-19

**Authors:** Bruno Wichmann, Roberta Moreira Wichmann, Tiago Almeida de Oliveira, Crysttian Arantes Paixão

**Affiliations:** a University of Alberta, Department of Resource Economics & Environmental Sociology, 503 General Services Building. Edmonton, AB, T6G-2H1, Canada; b Brazilian Institute of Education, Development and Research–IDP, Economics Graduate Program, SGAN 609, Brasília, DF, 70830-401, Brazil; c State University of Paraiba, Department of Statistics, Rua das Baraunas, 351, Bodocongó, Campina Grande, PB, 58429-500, Brazil; d Federal University of Bahia, Institute of Science, Technology and Innovation, Rua do Telégrafo, S/N, Centro, Camaçari, BA, 42809-000, Brazil

**Keywords:** Latitude, Longitude, Brazilian clinics, Postal codes, Postal code area, CEP, SUS

## Abstract

We develop a geocoded dataset of primary health care clinics in Brazil. We merge data from three publicly available sources. The first is the National Registry of Healthcare Facilities (CNES-ST), which collects the location (state, municipality, and 8-digit postal code) of all health care facilities, public or private, operating in Brazil. The second is the National Registry of Addresses for Statistical Purposes (IBGE-CNEFE), which contains the geographic coordinates of all addresses in Brazil (including 8-digit postal codes) and serves as the basis for the Brazilian census. Our approach aggregates individual (address-level) coordinates to the 8-digit postal code, and assigns coordinates to primary care clinics based on each clinics’ postal code. Using data from a third source, the IBGE shapefiles, we estimate the area of postal codes to evaluate the precision of our geo-referencing method. The unique facility identification number (cnes number) can be used to merge our georeferenced data with other publicly available databases of the Brazilian Unified Health System. The final dataset is an unbalanced panel with monthly observations about 293,698 primary care clinics’ locations (i.e. coordinates), from January 2018 to December 2023, totalling 15,455,219 observations.

Specifications Table

**Table:** 

Subject	Public Health and Health Policy.
Specific subject area	Spatial distribution of primary care medical clinics*.*
Type of data	Raw, Filtered, and Processed.
Data collection	Data were collected from three publicly available sources. First, the National Registry of Healthcare Facilities of the Brazilian Unified Health System (CNES-ST). Second, the National Registry of Addresses for Statistical Purposes of the Brazilian Institute of Statistics and Geography (IBGE-CNEFE). Third, shapefiles from the Brazilian Institute of Statistics and Geography (IBGE).
Data source location	Country: Brazil
Data accessibility	Repository name: Mendeley Data Data identification number: DOI: 10.17632/mct3prb32k.1 Direct URL to data: https://data.mendeley.com/datasets/mct3prb32k/1 Access the data and codes in the Mendeley Data repository. Download the compressed ‘Replication’ folder. Refer to the 'ReadMe' file for additional details.
Related research article	None.

## 1 Value of the Data

• To the best of our knowledge, the database is the first to attach geographic coordinates (latitude and longitude) to primary care facilities in Brazil. The raw data includes the universe of medical facilities registered to operate in Brazil. After filters and data processing, including imputation, the dataset provides coordinates to 99.4 % of primary care clinics operating in all 26 Brazilian States and the Federal District.
• Primary health care is the backbone of healthcare systems. The information about coordinates of individual primary care clinics allows for detailed spatial analyses of primary care in Brazil. For instance, the geocoded data allow researchers to examine the location of the Brazilian primary care infrastructure relative to the demand, e.g. distribution of population throughout the national territory. The final dataset can also be used to investigate the effects of access to primary healthcare services, and is particularly valuable for evaluating the walk-ride-walk travel chain in public transportation systems.
• The clinic identification number (cnes number) allows researchers to merge the clinics’ geographic coordinates offered in this paper with other clinics’ characteristics from the various (non-geocoded) databases of the Brazilian Unified Health System – SUS. These include databases from the System of Ambulatory Information (SIA-SUS), the Brazilian Vital Statistics (SINASC), and various databases from the National Registry of Healthcare Facilities, e.g. CNES-PF (professionals), CNES-EQ (equipment), or CNES-LT (beds). These data are available online at https://datasus.saude.gov.br/transferencia-de-arquivos.
• The information in the dataset is recorded in monthly files from January 2018 to December 2023, which allows for spatial and temporal examination of entries into and exits from the health care system. Moreover, the sample includes periods before, during, and after the COVID-19 pandemic. As such, the data allow for spatial analyses of the impact of the pandemic on primary care in Brazil.
• The data also offers new spatial information by developing an approach to use area measurements of the census sectors to approximate the area of postal codes. The size of a postal code can be used to evaluate the precision of our geo-referencing method.

## 2 Background

The Brazilian Unified Health System (SUS) is among the largest public healthcare systems in the world, with a mandate to provide all levels of care, free of charges or co-pays, to >200 million Brazilians [1]. Every health care facility in Brazil, privately or publicly managed or owned, is required by federal law to register with SUS [2]. The information provided is stored in a SUS database, namely: the National Registry of Healthcare Facilities (CNES). CNES holds several administrative datasets that describe facilities’ characteristics (CNES-ST), professionals and hours worked (CNES-PF), beds (CNES-LT), equipment (CNES-EQ), among others (see http://cnes.datasus.gov.br/). Each facility has a unique identification number (‘cnes’ number) that can be used to track facilities across different SUS databases.

The CNES-ST files contain important information about the location of healthcare facilities. Specifically, the database informs the facility’s state, municipality, and 8-digit postal code. However, geographic coordinates are not available. This is a significant shortcoming as coordinates allow for the use of Global Positioning Systems (GPS) tools that can facilitate detailed spatial analyses. This paper contributes by building a dataset that geo-references primary care medical clinics in the CNES-ST database [3].

## 3 Data Description

The objective of the paper is to assign a geographic coordinate to each primary care clinic in the CNES-ST database. To accomplish this goal, we use publicly available data from three sources. The first is the SUS medical facility database, CNES-ST. The second is the georeferenced address database from the Brazilian Institute of Statistics and Geography (IBGE), namely: the National Registry of Addresses for Statistical Purposes (CNEFE). The IBGE-CNEFE files list all addresses (including 8-digit postal codes) and their corresponding coordinates in the Brazilian national territory. As we describe in detail below, the paper aggregates coordinates information to the postal code level, and uses postal codes to merge these coordinates with data from individual primary care medical clinics in the CNES-ST database. The third data source is the Brazilian Institute of Statistics and Geography (IBGE). We collect IBGE’s shapefiles to construct a measurement of a postal code’s size (area in square km) to evaluate the precision of our geo-referencing approach.

All the data and codes used in this process, along with the final geo-referenced dataset produced by our approach, is available in the following Mendeley data depository: https://data.mendeley.com/datasets/mct3prb32k/1 [3]. The materials are included in a compressed folder entitled “Replication”. Inside, there are three folders and six Stata do files. The ‘RawData’ folder contains original .csv files (from CNES-ST and IBGE-CNEFE). It also contains a .dbf file with the area of census sectors (from IBGE), which we use to estimate the size of a postal code. The ‘Ancillary’ folder simply houses temporary files used in intermediate steps of data construction. The ‘FinalData’ folder contains the final product, the file ‘GeocodedPrimaryCareData’ with is available in .dta and .csv format. The six .do files clean and process the original raw files to construct the final dataset. The files should be executed in consecutive order, starting from Code1.do until Code6.do. Table 1 describes the twelve variables included in the final dataset.

**Table 1:** Dataset variables and their descriptions.

Variable Name	Description
state	The initials of the Brazilian State where the clinic is located
state_id	The IBGE code of the Brazilian State where the clinic is located
ibge	The IBGE code of the Municipality where the clinic is located
cnes	This is the clinic’s identification number
year	Year of the record
month	Month of the record
cep	8-digit postal code of the clinic
cep_area⁎	Estimate of the area of 8-digit postal code of the clinic (square km)
latitude⁎	Latitude of the clinic (decimal)
longitude⁎	Longitude of the clinic (decimal)
imputation⁎	=1 if missing postal code information was imputed
approximation⁎	=1 if coordinates come from 5-digit (instead of 8-digit) approximation of postal code coordinates

*Notes*: * Refer to the discussion below for details.

**Fig. 1:**
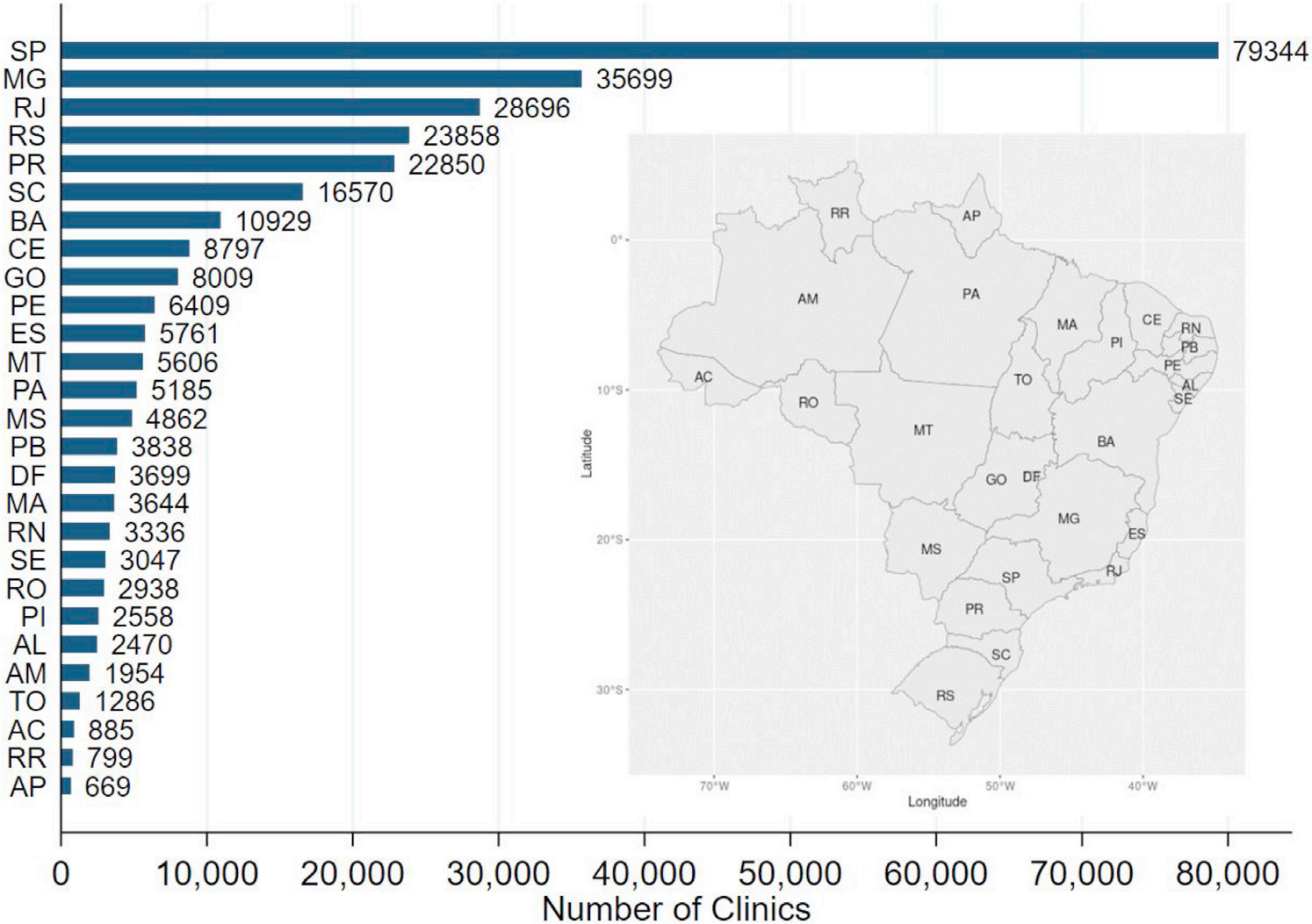
The distribution of primary care clinics, by State (*n* = 293,698).

An observation in the final data represents a clinic-period record. An observation indicates that the primary care clinic was operational at that time period (see details in the section below). As individual clinics may leave or enter the health system, the dataset is an unbalanced panel. The panel has 293,698 clinics and 72 time periods (monthly records from January 2018 to December 2023). In total, there are 15,455,219 observations. Table 2 shows the temporal distribution of the data. Fig. 1 shows how the 293,698 medical clinics are distributed across Brazilian States.

**Table 2:** Number of observations per period.

		Year						Total
		2018	2019	2020	2021	2022	2023	
**Month**	**1**	198,530	208,193	216,657	204,684	219,448	228,350	1,275,862
	**2**	199,241	209,165	207,900	206,564	220,448	228,038	1,271,356
	**3**	199,925	209,756	199,811	207,999	221,386	228,724	1,267,601
	**4**	200,935	210,800	200,424	209,500	222,188	229,786	1,273,633
	**5**	201,905	212,124	201,314	210,903	223,384	230,779	1,280,409
	**6**	202,668	212,827	196,605	212,039	224,152	232,221	1,280,512
	**7**	203,551	213,796	198,640	213,674	224,942	233,155	1,287,758
	**8**	204,275	213,189	199,546	214,935	226,028	234,315	1,292,288
	**9**	205,149	214,363	200,628	215,936	227,041	235,039	1,298,156
	**10**	206,171	215,320	201,909	217,034	228,049	235,986	1,304,469
	**11**	206,932	216,139	202,855	218,095	228,826	237,147	1,309,994
	**12**	207,481	216,405	203,496	218,611	229,461	237,727	1,313,181
**Total**		2,436,763	2,552,077	2,429,785	2,549,974	2,695,353	2,791,267	15,455,219

## 4 Experimental Design, Materials and Methods

### 4.1 Primary care clinics data (CNES-ST)

We download the facility (healthcare establishment) CNES-ST raw files from SUS’s website (https://datasus.saude.gov.br/transferencia-de-arquivos/). The files have the following naming format STXXYYMM.csv. Table 3 offers an explanation of the file names in the CNES-ST database. ST stands for establishment (the type of CNES file). The digits XX represents the initials (acronym) of the federal unit where the facility operates (either one for the 26 states or the federal district). The digits YY represent the last two digits of the year, from 2018 to 2022. The digits MM corresponds to two digits of the month of the year. As such, the ‘*STCE2001*.csv’ file corresponds to data from the state of Ceará (CE) from January 2020. In total, 1944 (27 states × 6 years × 12 months) files are downloaded and available in the RawData folder (see discussion above).

**Table 3:** Naming format of .csv files from CNES-ST.

File STXXYYMM.csv			
*XX* (Federal Unit)	*YY* (Year)	*MM* (Month)	Sample
AC, AL, AM, AP, BA, **CE**, DF, ES, GO, MA, MG, MS, MT, PA, PB, PE, PI, PR, RJ, RN, RO, RR, RS, SC, SE, SP, TO	18 19 **20** 21 22	**01** 02 03 04 05 06 07 08 09 10 11 12	ST**CE2001**.csv

The .csv files are converted into .dta format to be processed using Stata17. Collectively, the original files contain 25,377,928 records. To focus on records of primary healthcare clinics, we drop 9051,603 records that correspond to other types of healthcare facilities (e.g. hospitals, emergency rooms, pharmacies, specialty clinics, laboratories, etc.). Specifically, the observations kept in the sample correspond to those with facility type classification (TP_UNID) equal to 01, 02, 04, 15, 22, or 72.^1^ We also drop an additional 6383 observations that correspond to inactive primary care facilities, i.e. those who indicate no services were provided (variable ATEND_PR equals 0). Moreover, we keep only clinics that do not require a patient referral as referrals are typically required for specialized/secondary care (keep if variable CLIENTEL is equal to 01 or 03). As a result, an additional 778,811 observations are deleted.

At this stage, the sample has 15,541,131 observations and 70,065 unique 8-digit postal codes from 295,491 clinics. Approximately one percent of the observations (186,170) had missing postal code data. The missing postal code information is a limitation because the postal code is the variable used to geocode the position of medical clinics. We applied imputation methods to the postal code variable in order to maximize the size of the data geocoded data. The dataset contains a binary variable that indicates whether the postal code information for any particular clinic-month observation was originally reported or imputed.

The imputation method is as follows. As explained above, the CNES-ST data is reported monthly. 15,856 clinics (or 5.4 % of the clinics) did not report, at a point in time, postal code information. For those observations, we replace the missing postal code with the within-year mode postal code, i.e. for each clinic, the most frequently reported postal code in the year of the missing postal code. This allow us to recover 73,374 of the 186,170 missing postal code observations. For the remaining 112,796 observations, we replace missing postal code information with each clinic’s mode of the entire sampling period. This allow us to recover another 99,700 observations. Finally, the remainder 13,096 observations (or 0.08 % of the clinic-month records) cannot be geo-referenced via postal code and therefore are dropped from the sample. Below, in the ‘Limitations’ section, we discuss possible misrepresentations of locations due to the imputation process.

The final non-georeferenced CNES-ST sample contains 15,528,035 observations of 294,907 unique primary care clinics. This file is called Data1.dta and is available in the Ancillary folder.

### 4.2 Postal codes coordinates data (IBGE-CNEFE)

On May 21, 2024 the Brazilian Institute of Statistics and Geography (IBGE) released the National Registry of Addresses for Statistical Purposes (CNEFE) microdata to the general public (available online at: https://www.ibge.gov.br/estatisticas/sociais/populacao/38734-cadastro-nacional-de-enderecos-para-fins-estatisticos.html). The IBGE-CNEFE database contains georeferenced addresses for the entire country and serves as the basis for the 2022 Demographic Census. Specifically, the database includes geographic coordinates (latitudes and longitudes) for both residential and non-residential addresses (e.g., businesses, farms, schools, constructions, churches or temples, etc.).

The data is available in 27 (state) .csv files with the following naming format NN_XX.csv. Table 4 offers an explanation of the file names in the IBGE-CNEFE database. The digits NN represent the state’s numeric IBGE code and XX represents the initials. For instance, the ‘23_CE.csv’ file has data from all addresses in the State of Ceará (CE). In total, the IBGE-CNEFE contains information on 110,639,832 addresses, of which 90,358,192 are residential.

**Table 4:** Naming format of .csv files from IBGE-CNEFE.

File NN_XX.csv		
*NN* (state’s IBGE code)	*XX* (state’s initials)	Sample
12, 27, 13, 16, 29, **23**, 53, 32, 52, 21, 31, 50, 51, 15, 25, 26, 22, 41, 33, 24, 11, 14, 43, 42, 28, 35, 17	AC, AL, AM, AP, BA, **CE**, DF, ES, GO, MA, MG, MS, MT, PA, PB, PE, PI, PR, RJ, RN, RO, RR, RS, SC, SE, SP, TO	**23**_**CE**.csv

We use the universe of addresses (residential and non-residential) to aggregate the coordinates from street address (or unit) level to postal code level. Specifically, for every 8-digit postal code, we compute the (frequency weighted) average latitude and longitude. Our final geocoded postal code dataset contains of 928,941 observations. This file is called Data2.dta and is available in the Ancillary folder.

### 4.3 Estimating the area of a postal code

Our approach is to use each clinic’s 8-digit postal code, or CEP (Postal Addressing Code), to approximate the geographic coordinates of each medical clinic. This is possible because the structure of CEP is informative of the clinic’s location. The Brazilian CEP represent contiguous regions. Each of the first five digits of the postal code identifies with higher precision a subset of the geographic space of the contiguous region. The first digit of the postal code represents a Brazilian postal region. The second digit identifies a sub-region. The third identifies a sector. The fourth identifies a subsector. The fifth digit identifies a subsector divider. The last three digits are called distribution identifiers, or ‘postal suffix’. These three suffix digits server to further pinpoint the location of an address [4].

In urban areas, a distinct postal code may be assigned to a small geographic area, like a short street. However, in rural areas, a postal code may represent a larger area or an entire municipality. To the best of our knowledge, shapefiles of the geometry of Brazilian postal codes are not available. However, the IBGE has a shapefile for the Brazilian census sectors, which are the smaller geographic units used to plan and design the Brazilian census (available online at https://www.ibge.gov.br/geociencias/organizacao-do-territorio/estrutura-territorial/26565-malhas-de-setores-censitarios-divisoes-intramunicipais.html?t=acesso-ao-produto). Importantly, the areas of census sectors are available in the attribute table (.dbf file entitled ‘BR_Malha_Preliminar_2022.dbf, in the RawData folder). Census sectors divide the national territory into continuous areas. The median area of a census sector is 0.13 km^2^.

Our geo-referring method can be thought of as a calculation of coordinates of the centroid of postal codes. To better understand the precision of this approach, we use the area of the Brazilian census sectors to approximate the size of the geographic area spanning a postal code. Our geocoded addresses database (IBGE-CNEFE) includes, in addition to the postal code and coordinates of each address, the census sector in which an address is located. We use the census sector to merge the postal codes in the IBGE-CNEFE with census sectors’ areas from the shapefiles. We note, however, that there is not a one-to-one correspondence between postal codes and census sectors. These two geographic spaces overlap such that a postal code can span multiple census sectors and a census sector can overlap with multiple postal codes.

The information in the ‘BR_Malha_Preliminar_2022.dbf’ file allows us to calculate the country’s area of 8510,418 km^2^. While census sectors cover the entire national territory, postal codes are not assigned to all areas of Brazil. The match between the shapefile (.dbf file with census sectors’ areas) and the postal codes (from IBGE-CNEFE) allows us to measure the size of remote areas of the national territory, i.e. those without a postal code or a clinic. After matching postal codes and census sectors, we find that the postal code coverage correspond to 87.03 % of the Brazilian national territory (7406,825 km^2^).

We proceed to split these 7406,825 km^2^ into postal codes by simply dividing the census sector area by the number of postal codes in each sector. We then collapse the data by summing all the average sector’s areas to the postal code level. While we acknowledge that this approach offers only a simple approximation of the area of 8-digit postal codes, it has the advantage of not ‘double counting’ the national territory. We find that the distribution of 8-digit postal code size in our sample is significantly skewed to the right. The skewness test rejects the null of normality with *p* < 0.001.On average, a postal code spans an area of 95.19 km^2^. The 25th, 50th (median), and 75th percentiles are 0.017, 0.035, and 0.083 km^2^, respectively.

The estimates of a postal codes’ areas allow us to better understand how far the centroid of a postal code is from its boundary. That is, postal code areas can be used to gauge approximations errors of using postal codes’ centroids to pinpoint clinics’ locations. For example, assuming a postal code has a circular shape, and that the centroid coordinate is located at the center of the circle, the radius of the circle represents the maximum geocoding error (measured in straight line km). Table 5 provides estimates of these errors along the distribution of postal codes sizes. For the median clinic, our estimate of the maximum geocoding error is 110 m Assuming clinics are uniformly spatially distributed within the circle, the expected geocoding error is equal to 2*radius/3. For the median postal code size, the expected geo-referencing error is approximately 73 m In general, the data in Table 5 suggests that, for the vast majority of clinics, using postal codes to geo-reference their position results in relatively precise estimates of their locations.

**Table 5:** Maximum geocoding error along the distribution of postal code size.

Postal Code Size (square km)	Maximum Geocoding Error (km)
0.009 (10th percentile)	0.055
0.017 (25th percentile)	0.074
0.035 (median)	0.110
0.083 (75th percentile)	0.160
10.421 (90th percentile)	1.820

### 4.4 Geo-referencing primary care clinics: merging CNES-ST and IBGE-CNEFE datasets

We use 8-digit postal codes to merge the pre-processed CNES-ST data (the non-geocoded clinic data in the file Data1.dta, discussed above) with the pre-processed IBGE-CNEFE data (geocoded postal code data in the file Data2.dta, discussed above). There are 864,675 observations that represent geocoded postal codes in the IBGE-CNEFE data that are not needed as there are no primary care medical clinics in these postal codes.

Out of the 15,528,035 clinic-month observations, 894,469 have 8-digit postal codes that do not match an 8-digit postal code in the official IBGE-CNEFE file. On average, this corresponds to 5.6 % of the observations at the state-level, and 2.4 % at the municipality-level. These observations likely represent errors in the reporting of 8-digit postal codes by clinics in the CNES-ST database. For these observations, we use 5-digit postal codes to approximate the geographic coordinates and merge the two files. Specifically, the steps to merge the data are as follows. First, we compute a cep5 variable (first 5-digits of the full postal code) in the CNES-ST data. Second, we repeat this step and compute a cep5 variable in the address level IBGE-CNEFE file. We then aggregate coordinates by computing their (frequency weighted) averages for each 5-digit postal code. In summary, the 894,469 observations without direct (8-digit) postal code match correspond to 5804 8-digit postal codes or 3091 5-digit postal codes.

Finally, exclusively for the observations without matches, we use the 5-digit postal code to merge the CNES-ST and the IBGE-CNEFE. This approach allows us to match 855,487 (out of the 894,469) observations. Again, we flag these observations in the final dataset by including a binary variable that indicates whether the coordinates for a clinic-month observation comes from an 8-digit or 5-digit postal code approximation.

After the 5-digit approximation, there are still 1209 clinics that, at some point in the sampling period, did not have postal code information that allows for geo-referencing. We drop these 1209 clinics (only 0.4 % of total number of clinics). This leads to dropping 72,816 observations.

This concludes our approach. Our final sample has 15,455,219 georeferenced clinic-month records, from 293,698 clinics. These data are in the file GeocodedPrimaryCareData in the FinalData folder.

## Limitations

The imputation method may generate geo-referring errors. For example, consider a clinic that relocates in November of a given year and assume there is missing postal code information for December. The imputation approach will replace the missing December postal code with the mode postal code, i.e. the postal code from January to October, which would misrepresent the location of the clinic in December.

Several important remarks are in order that minimize potential concerns regarding our imputation method. First, the data contains an imputation indicator, which allows data users to dismiss the imputed geo-position if deemed appropriate, or create alternative imputation criteria (e.g. last known location). Second, as discussed above, only a small fraction of the observations (1.2 %) are subject to imputation. Third, a significant share (42 %) of the medical clinics that are (at some point in time) subject to postal code imputation has fixed (unchanged) postal code information throughout the sampling period. These cases likely represent correctly imputed locations. To see why, note that an imputation resulting in fixed location would only represent an incorrect position if the clinic were to move to a different location for a short period of time (less than the mode) and not report a single postal code in the new location. Fourth, relocation of a medical clinic is a rare event. Using the sample of clinics for which we have complete location information, we find that only 4.76 % have (at some point) changed geographic positions. Fifth, relocations across municipality borders would not be misrepresented in our data as practices that move across municipality lines are likely to receive a different identification (cnes) number. As a result, possible geographic discrepancies would be limited to municipality borders. Sixth, and finally, for the clinics that at some point did change locations, we compute the variance in coordinates (latitude and longitude) and test the difference of the mean variance between the group that received imputation and the group that did not. As municipalities are responsible to manage public primary care delivery in Brazil, we cluster the standard errors of the test at the municipality level to allow for within primary care system correlation between the observations. The results indicate that we cannot reject the null (at 10 % significance level) that the means of the variance in coordinates between the two groups (imputed and not imputed) are the same. This result suggests that, for the group of clinics that relocate, the variance of their positions are, on average, the same between the imputed and not imputed groups of clinics. Simply put, the variance in positions we observe in the data is not a function of the imputation process.

## Ethics Statement

DATASUS provides public and anonymous data, in compliance with Article I of Resolution 510/2016 of the National Research Ethics Commission [5]. The authors have read and followed the ethical requirements for publication in Data in Brief. The authors confirm that the current work does not involve human subjects, animal experiments, or any data collected from social media platforms*.*

## CRediT Author Statement

**Bruno Wichmann**: Conceptualization, Methodology, Software, Validation, Formal analysis, Investigation, Writing –original draft. **Roberta Moreira Wichmann**: Data curation, Writing –review & editing, Supervision, Project administration. **Tiago Almeida de Oliveira**: Methodology, Software, Writing –review & editing. **Crysttian Arantes Paixão**: Methodology, Software, Writing –review & editing.

## Data Availability

Mendeley DataData and codes to produce a geocoded dataset of primary health care clinics in Brazil (Reference data)

## Footnotes

1 We note that the code can be easily adapted to include other types of facilities. For example, hospitals and emergency rooms can be incorporated by retaining observations where the facility type classification variable (TP_UNID) is equal to 05 and 20, respectively.
